# Proteome and Acetylome Analysis Identifies Novel Pathways and Targets Regulated by Perifosine in Neuroblastoma

**DOI:** 10.1038/srep42062

**Published:** 2017-02-06

**Authors:** Xiao Gu, Zhongyan Hua, Yudi Dong, Yue Zhan, Xiaowen Zhang, Wei Tian, Zhihui Liu, Carol J. Thiele, Zhijie Li

**Affiliations:** 1 Medical Research Center, Shengjing Hospital of China Medical University, Shenyang, 110004, China; 2 Cellular & Molecular Biology Section, Pediatric Oncology Branch, National Cancer Institute, National Institutes of Health, Bethesda, Maryland, 20892, USA

## Abstract

Perifosine, an Akt inhibitor, has been shown to be effective in controlling neuroblastoma tumor growth. However, studies indicate that in addition to the ability to inhibit Akt, other mechanisms contribute to perifosine’s anti-tumor activity. To gain insight into perifosine anti-tumor activity in neuroblastoma we have studied changes in the proteome and acetylome after perifosine treatment in SK-N-AS neuroblastoma cells using SILAC labeling, affinity enrichment, high-resolution and LC-MS/MS analysis. Bioinformatic analysis indicates that, a total of 5,880 proteins and 3,415 lysine acetylation sites were quantified in SK-N-AS cells and 216 differentially expressed proteins and 115 differentially expressed lysine acetylation sites were obtained. These differentially expressed proteins and lysine acetylated proteins were involved in a number of different biological functions, metabolic pathways and pathophysiological processes. This study details the impact of perifosine on proteome and lysine acetylome in SK-N-AS cells and expands our understanding of the mechanisms of perifosine action in neuroblastoma.

Neuroblastoma (NB) is the most common extracranial solid tumor in childhood. Reminiscent of its neural crest origin^1^, tumors present along the sympathetic chain, especially in the adrenal medulla or paraspinal ganglia^2^. After leukemia and brain tumors it is the third leading cause of pediatric cancer. Although its incidence rate is only 7–8% of pediatric malignancies, the mortality rate accounts for approximately 15% of all pediatric cancer deaths. According to its clinical manifestation and biological characteristics, NB patients can be divided into low, intermediate and high-risk groups^3^. Low and intermediate risk NB patients can benefit from surgery and chemotherapy with long-term survival rates up to 90%. Unfortunately, high-risk NB patients still have a poor prognosis and their long-term survival rate remains <50% in spite of increasingly intensive treatment strategies, such as high-dose chemotherapy, radiotherapy, and bone marrow transplantation^4^. Unlike other pediatric malignancies, the cure rate and long-term survival rate of high-risk NB patients have not significantly improved with the progress of detection technologies and treatment approaches^5^,^6^. Therefore, new therapeutic approaches and better understanding of tumor response to treatment are needed to improve treatment efficacy in high-risk NB patients.

Akt activation is detected in the tumor tissues of patients with high-risk NB, and is a predictor of poor prognosis^7^. Such studies indicate that Akt may be a potential therapeutic target for NB. Perifosine, an alkylphospholipid Akt inhibitor, inhibits Akt activation by preventing the recruitment of Akt to cell membrane^8^,^9^,^10^. Our previous studies showed that perifosine, as a single agent, inhibited the NB growth *in vitro* and *in vivo*, as well as improved the sensitivity of NB to chemotherapeutic drugs^11^,^12^. Similar results were found in other tumors^13^,^14^,^15^,^16^. Mechanistically, besides blocking Akt, perifosine has been found to exert its anti-tumor growth effect through inhibition of MAPK pathway^17^, reduction of telomerase activity and shortening of telomeres^13^, induction of reactive oxygen species (ROS) production and phosphorylation of JNK and P38^18^ and inhibition of phosphatidylcholine (PC) synthesis^19^.

In this study, to explore the mechanisms underlying perifosine treatment, the global proteome and lysine acetylome profile in SK-N-AS (AS) cells after perifosine treatment were intensively studied by a combination of SILAC labeling, affinity enrichment, high-resolution LC-MS/MS analysis and intensive bioinformatic analysis (Fig. 1). We obtained 216 differentially expressed proteins from the 5,880 quantified proteins, and 115 differentially expressed lysine acetylation sites from the 3,415 quantified lysine acetylation sites. These proteins mainly exist in the nucleus and cytoplasm, and are involved in a wide variety of metabolic pathways and biological functions, such as lipid metabolic process, apoptosis and transcription. We explored the mechanisms underlying perifosine treatment and our results provided novel insights into the action and acetylation caused by perifosine.

## Results

### Inhibition of cell survival and Akt phosphorylation by perifosine in AS cells

AS cells were treated with different concentrations of perifosine (2.5, 5, 7.5, 10, 15, 20, 30, 40, 50 and 60 μM) for 48 h and MTS assay was used to detect cell survival. Perifosine induced a concentration-dependent decrease in AS cell survival (Supplementary Fig. S1a). We treated AS cells with varying concentrations (2.5, 5, 7.5, 10 and 15 μM) of perifosine for 16 h to study the effect of perifosine on the expressions of phosphorylated-Akt and protein acetylation. We found that Akt phosphorylation was inhibited by perifosine at the five concentrations we tested (Supplementary Fig. S1b), while 10 μM of perifosine induced the most significant changes in protein acetylation (Supplementary Fig. S1c). So we used 10 μM of perifosine for the following proteome and acetylome analysis.

### Impacts of perifosine treatment on global proteome level in AS cells

Changes in the global proteome of AS cells in response to perifosine treatment were analyzed. In total, 6,731 proteins were identified, among which 5,880 proteins were quantified. By setting quantification ratio of >1.5 as up-regulated threshold and <0.67 as down-regulated threshold, we detected 124 up-regulated proteins and 92 down-regulated proteins after perifosine treatment.

**Figure 2:**
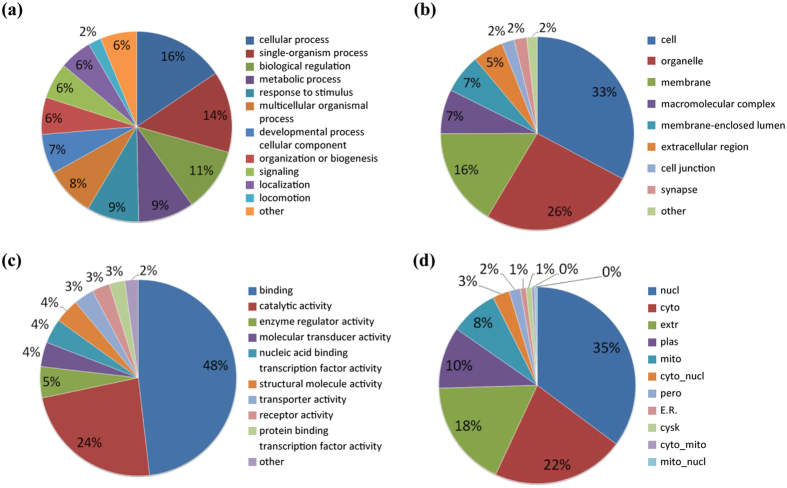
GO and subcellular location annotation of the proteome in perifosine-treated AS cell line. (**a**) biological process, (**b**) molecular function, (**c**) cellular component, (**d**) subcellular location.

To characterize the functions and subcellular locations of the differentially expressed proteins in response to perifosine treatment, bioinformatic analysis on Gene Ontology (GO) and subcellular functional annotation were carried out. The GO annotation analysis included biological process, cellular component, and molecular function. For the biological process classification in GO annotation, the largest group was the proteins associated with the cellular process (16%), followed by single-organism process (14%), biological regulation (11%), metabolic process (9%), and response to stimulus (9%) (Fig. 2a). The top three lists of the cellular components were cell (33%), organelle (26%) and membrane (16%) (Fig. 2b). The altered proteins involved in binding (48%) and catalytic activity (24%) accounted for the main proportion of proteins enriched by molecular function analysis (Fig. 2c). The differentially expressed proteins were mainly distributed in the nucleus (35%), cytoplasm (22%) and extracellular region (18%) in the subcellular location category (Fig. 2d).

**Figure 3:**
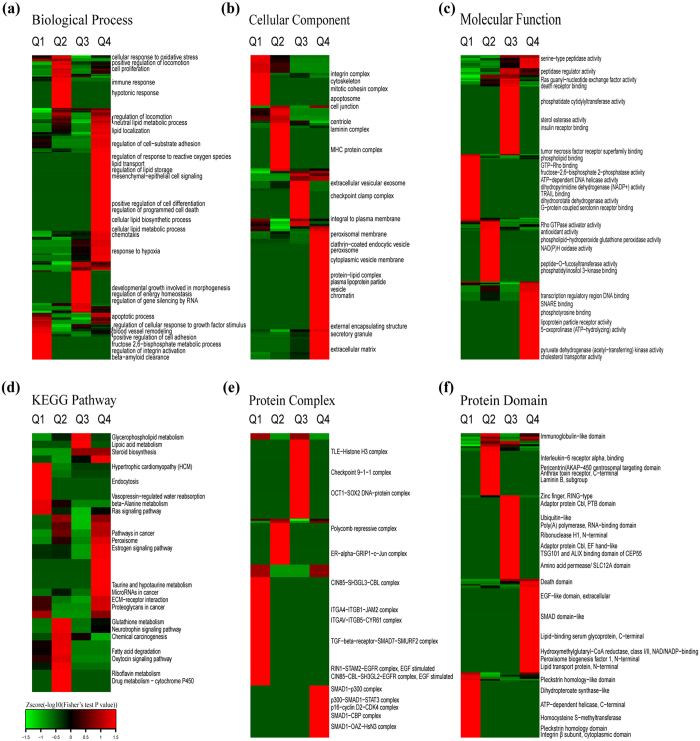
Enrichment-based clustering analysis of the proteome in perifosine-treated AS cell line. (**a**) Biological process, (**b**) molecular function, (**c**) cellular component, (**d**) KEGG pathway, (**e**) protein complex, (**f**) protein domain. In each classification, all the quantified proteins were divided into four groups according to L/H ratios: Q1 (Ratio < 0.67), Q2 (0.67 < Ratio < 0.77), Q3 (1.3 < Ratio < 1.5), Q4 (Ratio > 1.5).

To reveal the nature of the quantified proteins after perifosine treatment in AS cells, GO enrichment-based clustering analysis was performed (Fig. 3a–c). All the quantified proteins were divided into four groups according to quantification ratios: Q1 (Ratio < 0.67), Q2 (0.67 < Ratio < 0.77), Q3 (1.3 < Ratio < 1.5), Q4 (Ratio > 1.5).

The biological process enrichment-based clustering analysis showed that several processes were enriched in the up-regulated proteins (Q4), including cellular lipid metabolic process, programmed cell death, negative regulation of cell proliferation, regulation of localization, regulation of cell-substrate adhesion, positive regulation of cell differentiation, mesenchymal-epithelial cell signaling, chemotaxis, and response to hypoxia. The processes enriched in the down-regulated proteins were regulation of cellular response to growth factor stimulus, fructose 2,6-bisphosphate metabolic process, β-amyloid clearance, and immune response (Fig. 3a).

In the cellular component category, up-regulated proteins were mainly distributed in extracellular, cytoplasmic vesicle, peroxisome, and plasma membrane. In contrast, down-regulated proteins were located on cytoskeleton, cell junction, laminin complex and MHC protein complex (Fig. 3b).

The molecular function enrichment-based clustering analysis indicated that transcription regulatory, DNA binding and SNARE binding were enriched in the up-regulated proteins. Functions enriched in the down-regulated proteins correlated with metabolism such as phospholipid binding, GTP-Rho binding, phosphatase activity, dihydropyrimidine dehydrogenase (NADP+) activity, dihydroorotate dehydrogenase, and ATP-dependent DNA helicase activity (Fig. 3c).

To identify cellular pathways, protein complexes and protein domains involved in perifosine treatment in AS cells, we performed clustering analysis based on KEGG pathway, protein complex and protein domain (Fig. 3d–f). All the differentially expressed proteins were separated into four groups (Q1, Q2, Q3 and Q4) as in the GO enrichment-based clustering analysis.

The dominant pathways enriched in the up-regulated proteins were microRNAs in cancer pathway and glycerophospholipid metabolism pathway. While β-alanine metabolism pathway and Ras signaling pathway were enriched in the down-regulated proteins (Fig. 3d).

The clustering analysis on “protein complex” showed that the complexes in the formation of SMAD1 (for instance, p300-SMAD1-STAT3 complex, SMAD1-CBP complex, SMAD1-OAZ-HsN3 complex, SMAD1-p300 complex) and p16-cyclinD2-CDK4 complex were enriched in the up-regulated proteins. While the TGF-, EGFR-, ITGAV-ITGB5-, ITGA4-ITGB1-, or CIN85- contained complexes, such as TGF-β-receptor-SMAD7-SMURF2 complex, RIN1-STAM2-EGFR complex (EGF stimulated), ITGA4-ITGB1-JAM2 complex, ITGAV-ITGB5-CYR61 complex, and CIN85-CBL-SH3GL2-EGFR complex (EGF stimulated), were enriched in the down-regulated proteins (Fig. 3e).

The clustering analysis on “protein domain” indicated that death domain, lipid transport protein (N-terminal), and EGF-like domain (extracellular) were enriched in the up-regulated proteins after perifosine treatment. While the pleckstrin homology domain, homocysteine S-methyltransferase and dihydropteroate synthase-like domain were enriched in the down-regulated proteins upon perifosine treatment in AS cells (Fig. 3f).

**Figure 4:**
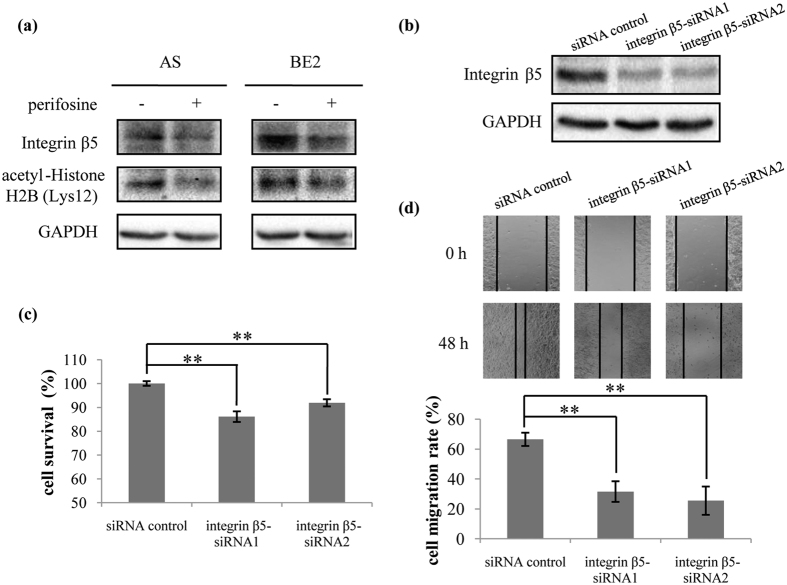
Validation of the global proteome and acetylome results and functional study of integrin β5. (**a**) Validation of the global proteome and acetylome results. AS and BE2 cells were treated with 10 μM of perifosine for 16 h. Total proteins were extracted and 30 μg of protein was analyzed for integrin β5 and acetyl-Histone H2B (Lys12) by western blotting. GAPDH was used as loading control. (**b**), (**c**) and (**d**) (**b**) AS cells were transfected with siRNA control, integrin β5-siRNA1 and integrin β5-siRNA2 for 48 h. Total proteins were extracted and 30 μg of protein was analyzed for integrin β5 protein by western blotting. GAPDH was used as loading control. (**c**) AS cells were transfected with siRNA control, integrin β5-siRNA1 or integrin β5-siRNA2. MTS assay was used to assess cell survival at 72 h of transfection. Means and standard deviations were shown. **p < 0.01, integrin β5-siRNA1 transfected cells or integrin β5-siRNA2 transfected cells vs. siRNA control transfected cells. (**d**) AS cells transfected with siRNA control, integrin β5-siRNA1 or integrin β5-siRNA2 were seeded into 24-well plates and scratched with a 200-μl pipette tip across the center of the well at 48 h of transfection, and then gap closing was photographed. The cell migration rate was calculated as described in “Methods” section. Bars, SD. **p < 0.01, integrin β5-siRNA1 transfected cells or integrin β5-siRNA2 transfected cells vs. siRNA control transfected cells.

We validated the results from the global proteome analysis by western blotting in two neuroblastoma cell lines, AS and BE2 cells. The expression of integrin β5 was down-regulated after 10 μM of perifosine treatment in both AS and BE2 cells (Fig. 4a), which was consistent with the quantitative results from global proteome analysis performed in AS cells. Based on the results of validation, we next performed integrin β5-siRNA transfection to further investigate the biological functions of integrin β5 in AS cells. The expression of integrin β5 protein was reduced after transfection of integrin β5-siRNA1 and integrin β5-siRNA2 into AS cells (Fig. 4b). The MTS and wound healing assay results suggested that knockdown of integrin β5 significantly induced cell death (*p* < 0.01) and inhibited cell migration (*p* < 0.01) (Fig. 4c,d). Our data indicated that knockdown of integrin β5 took the same effect as perifosine on AS cells survival and migration.

### Impacts of perifosine on acetylome level in AS cells

Changes on acetylome in AS cells in response to perifosine treatment were analyzed. In total, 3,468 lysine acetylation (Kac) sites in 1,417 proteins were identified, among which 3,415 sites in 1,398 proteins were quantified. By setting quantification ratio of >1.5 as up-regulated threshold and <0.67 as down-regulated threshold, we detected 56 up-regulated Kac sites in 50 proteins and 59 down-regulated Kac sites in 50 proteins.

**Figure 5:**
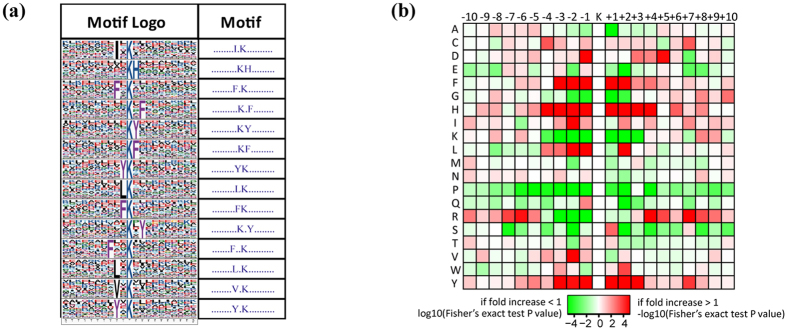
Motif analysis of the identified acetylation peptides. (**a**) sequence logo of acetylation motifs, (**b**) heat map of amino acid frequencies of the sequences flanking Kac sites.

To characterize the possible specific sequence motifs surrounding acetylated lysine residues in perifosine-treated AS cells, a motif analysis was generated to indicate the likelihood of amino acids being over- or under-represented at the positions surrounding the Kac sites (Fig. 5). Fourteen significantly enriched motifs were found from all of the identified Kac sites, including I*Kac, KacH, F*Kac, Kac*F, KacY, KacF, YKac, LKac, FKac, Kac*Y, F**Kac, L*Kac, V*Kac, and Y*Kac (* represents a random amino acid residue, Fig. 5a). The amino acid frequencies of the sequences flanking Kac sites were assessed to confirm whether there were position-specific amino acids adjacent to Kac sites by motif model (Fig. 5b). We found that phenylalanine acid (F), histidine acid (H) and tyrosine acid (Y) were overrepresented in multiple positions (±1, ±2, ±3) surrounding Kac sites. Isoleucine acid (I), leucine acid (L) and valine acid (V) were also overrepresented in the neighboring position of Kac. In addition, lysine acid (K), proline acid (P) and arginine acid (R) were frequently depleted at ±1, −2 position of Kac site, but arginine acid (R) preferred to appear in relatively distant positions such as +4 and +7 position of Kac site. Interestingly, the occurrence frequencies of phenylalanine acid (F), histidine acid (H) and tyrosine acid (Y) surrounding Kac were relatively high, while proline acid (P) surrounding Kac was usually low.

**Figure 6:**
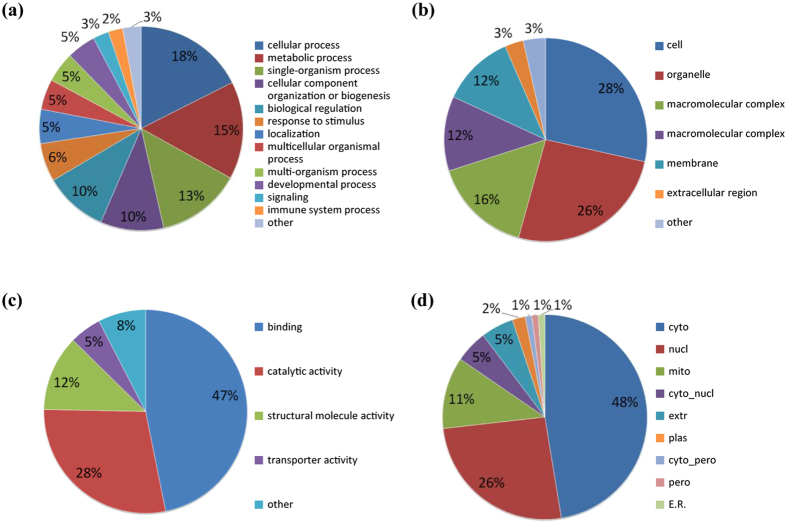
GO and subcellular location annotation of the acetylome in perifosine-treated AS cell line. (**a**) Biological process, (**b**) molecular function, (**c**) cellular component, (**d**) subcellular location.

To better understand the biological feature and functional alteration of lysine acetylome in response to perifosine treatment in AS cells, we performed the GO classification and subcellular location analysis (Fig. 6). In the biological process category, proteins were highly enriched in the cellular process (18%), metabolic process (15%) and single-organism process (13%) (Fig. 6a). The three principal cellular components were cell (28%), organelle (26%) and macromolecular complex (16%) (Fig. 6b). For the molecular function analysis, we found that the proteins related to binding (47%), catalytic activity (28%) and structural molecule activity (12%) were enriched (Fig. 6c). The subcellular location classification indicated that the differentially expressed Kac proteins were predominantly located in the cytoplasm, nucleus and mitochondria, which accounted for 47%, 26% and 11% respectively (Fig. 6d).

**Figure 7:**
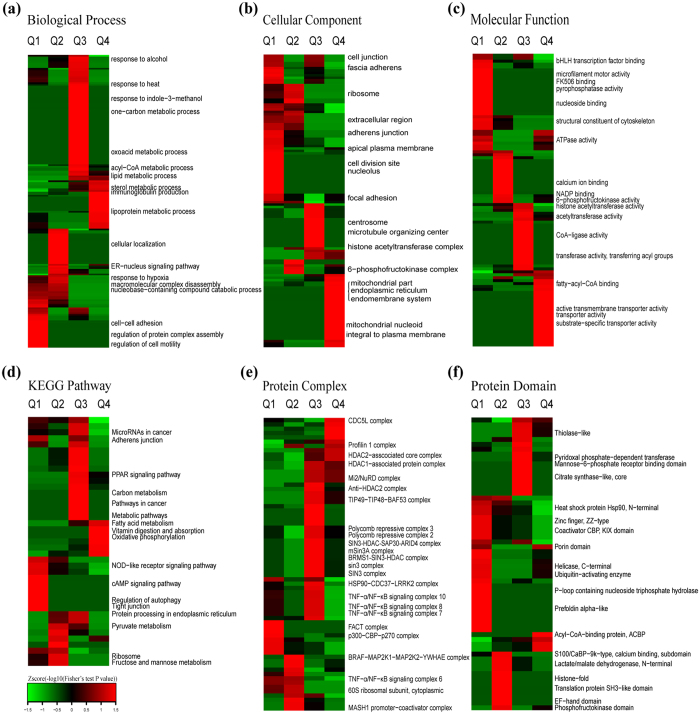
Enrichment-based clustering analysis of the acetylome in perifosine-treated AS cell line. (**a**) Biological process, (**b**) molecular function, (**c**) cellular component, (**d**) KEGG pathway, (**e**) protein complex, (**f**) protein domain. In each classification, all the quantified lysine acetylation proteins were divided into four groups according to L/H ratios: Q1 (Ratio < 0.67), Q2 (0.67 < Ratio < 0.77), Q3 (1.3 < Ratio < 1.5), Q4 (Ratio > 1.5).

To reveal the functional changes of protein acetylome regulated by perifosine in AS cells, we investigated the GO enrichment-based clustering analysis. All the quantified Kac proteins were divided into four groups (Q1, Q2, Q3 and Q4) according to quantification ratios described above (Fig. 7a–c).

On the biological process classification, metabolism-related and immunity-associated processes (such as lipid metabolic process, sterol metabolic process, lipoprotein metabolic process, and immunoglobulin production) were prominently enriched in the up-regulated Kac proteins (Q4). Meanwhile, processes associated with complex assembly and disassembly, cell adhesion and locomotion, and response to hypoxia were prominently enriched in the down-regulated Kac proteins (Fig. 7a).

Results of cellular component by category indicated that the acetylation level of the proteins involved in mitochondrial part, endoplasmic reticulum and endomembrane system was increased. The acetylation level of those proteins related to cell junction, ribosome, extracellular region and cell division site was decreased (Fig. 7b).

The molecular function enrichment-based clustering analysis indicated that proteins involved in fatty-acyl-CoA binding, transporter activity and active transmembrane transporter activity were intensively enriched in up-regulated acetylation level (Q4). However, proteins with down-regulated acetylation level were enriched in ATPase activity, pyrophosphatase activity, and nucleoside binding (Q1) (Fig. 7c).

To identify the cellular pathways, protein complex and protein domain regulated by perifosine in AS cells, we carried out clustering analysis based on KEGG pathway, protein complex and protein domain (Fig. 7d–f). All the quantified Kac proteins were divided into four groups (Q1, Q2, Q3 and Q4) as described above.

The KEGG pathway clustering analysis showed that the pathways of fatty acid metabolism, vitamin digestion and absorption, oxidative phosphorylation, microRNAs in cancer, protein processing in endoplasmic reticulum, pathways in cancer, and metabolic pathways were enriched in the up-regulated Kac proteins (Q3 and Q4). By contrast, the pathways enriched in the down-regulated Kac proteins were cAMP signaling pathway, regulation of autophagy, tight junction, pyruvate metabolism, and ribosome (Fig. 7d).

The clustering analysis on protein complex showed that the proteins associated with CDC5L complex, HDAC1-associated protein complex, Mi2/NuRD complex, polycomb repressive complex 2, SIN3 complex, HSP90-CDC37-LRRK2 complex, and TNF-α/NF-κB signaling complex were enriched in Q3 and Q4 with up-regulated Kac level. In contrast, FACT complex, p300-CBP-p270 complex, BRAF-MAP2K1-MAP2K2-YWHAE complex, and TNF-α/NF-κB signaling complex 6, all were enriched in down-regulated Kac proteins (Q1 and Q2) (Fig. 7e).

Results of protein domain clustering analysis indicated that acyl-CoA-binding protein (ACBP), porin domain, thiolase-like domain, and pyridoxal phosphate-dependent transferase were obviously enriched in Q3 and Q4 with increased Kac protein levels. On the contrary, heat shock protein Hsp90 (N-terminal), zinc finger (ZZ-type), EF-hand domain, and phosphofructokinase domain were distinctly enriched in Q1 and Q2 which had decreased Kac protein level (Fig. 7f).

We validated the results from the acetylome analysis in two neuroblastoma cell lines (AS and BE2). The expression of acetyl-Histone H2B (Lys12) was down-regulated after 10 μM of perifosine treatment in both AS and BE2 cells (Fig. 4a), which were consistent with the quantitative results of acetylome performed in AS cells.

### Protein-protein interaction network of lysine acetylome upon perifosine treatment in AS cells

**Figure 8:**
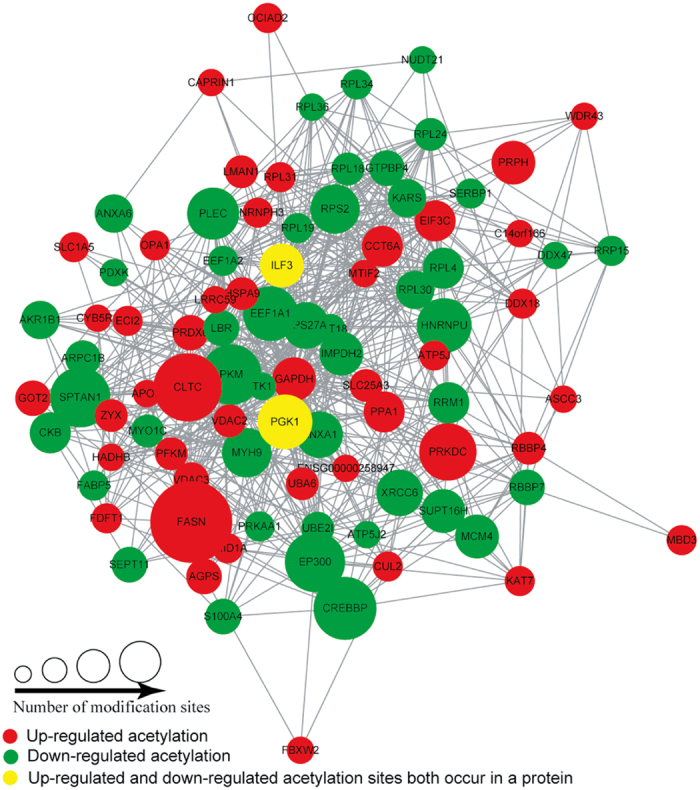
Protein-protein interaction network of acetylation proteins in perifosine-treated AS cell line.

To identify the chief nodes and important connectors among the Kac proteins after perifosine treatment in AS cells, we generated a protein-protein interaction network analysis based on the identified Kac proteins. There were 92 nodes and 762 interactions in protein-protein interaction network. The highest ten central nodes were GAPDH, EEF1A1, RPS27A, HSPA9, RPL4, PKM, EEF1A2, IMPDH2, RPS2 and EP300. We observed tight communications among the Kac proteins and highly connected clusters in ribosome (Fig. 8).

## Discussion

Previous studies have shown that Akt phosphorylation in tumor tissues is associated with the reduction of event-free survival and overall survival of NB patients^7^. Our previous studies using the allosteric Akt selective inhibitor MK-2206 showed little single agent activity in NB cell lines *in vivo*^20^. In contrast, the Akt inhibitor perifosine, as a single agent, inhibited NB growth in preclinical modes of NB^11^,^12^. To identify novel mechanism of perifosine action in NB cells, we investigated the changes in the proteome and lysine acetylome after perifosine treatment in AS cells. We obtained 216 differentially expressed proteins and 115 differentially expressed lysine acetylation sites in perifosine-treated AS cells. These proteins and sites were found predominantly in the nucleus and the cytoplasm, and involved in diverse biological regulation processes and cellular functions, including lipid metabolic process, integrins, apoptosis and transcription.

Studying the global proteome changes after perifosine treatment in AS cells, we found that many terms, which had been identified to be involved in perifosine actions in published studies were enriched. The cellular components involved in plasma lipoprotein particle and the biological processes involved in lipid biosynthetic, storage, transport, localization and metabolism were enriched. The molecular functions involved in lipoprotein particle receptor activity, cholesterol transporter activity and phospholipid binding were enriched. The pathways involved in glycerophospholipid metabolism and the protein domain involved in lipid transport were enriched. Perifosine has been reported to have marked effects on lipid metabolism, cholesterol and lipoprotein homeostasis^19^,^21^,^22^. Our study showed that pleckstrin homology domain (PH domain) was enriched in the down-regulated proteins of protein domain category. This result was consistent with the known mechanism of perifosine^9^.

It had been reported that perifosine induced ROS production^18^, and in our present study the biological processes related to ROS regulation and cellular response to oxidative stress were clearly enriched in global proteome changes. Furthermore, previous evidence had shown that perifosine induced cell apoptosis via up-regulation of TRAIL receptor expression and increased-recruitment of death receptors^23^,^24^,^25^. The biological processes associated with regulation of programmed cell death, the cellular component associated with apoptosome, the molecular functions associated with death receptor binding and TRAIL binding, and the protein domain associated with death domain were enriched in global proteome changes.

In the present study we identified a group of integrins, such as ITGAV-ITGB5-CYR61 complex and the ITGA4-ITGB1-JAM2 complex, which were noticeably down-regulated in the protein complex clustering analysis. Integrins transduce mechanical and chemical stimuli from the extracellular matrix (ECM) to intracellular signaling components. They play a role in cell adhesion, stemness, survival, metastasis and drug resistance^26^,^27^. Integrin αvβ5 was found in the majority of microvessels in Stage IV NB^28^ and blocking integrin αvβ5 significantly reduced tumor invasiveness and angiogenesis^29^,^30^. An inhibitor named cilengitide targeting both αvβ3 and αvβ5 integrins was approved effective on NB^31^. Up-regulation of integrin β1 was found to promote cell adhesion and resistance to chemotherapy^32^. Some agents inhibited cancer cell metastatic potential by down-regulation of integrin β1 expression or changes of its localization^33^,^34^. In our study, using clustering analysis, we found that some biological processes or cellular components were enriched in down-regulated proteins, such as integrin complex, integrin β subunit, cytoskeleton, regulation of integrin activation, positive regulation of cell adhesion and locomotion, and blood vessel remodeling. Our results suggested that knockdown of integrin β5 reduced the migration and survival in AS cells (Fig. 4b–d). These results provide the clue that blocking integrins is a potential mechanism for perifosine in the treatment of NB. Further experiments are needed to elucidate the mechanism of perifosine effect on integrins.

At acetylome level, there were a large number of metabolism-related processes enriched in the up-regulated Kac proteins on the classification of biological process with perifosine treatment. The result indicated a close relationship between Kac and metabolic regulation in tumor cells, which may help us further explore novel mechanisms of perifosine. Lysine acetylation is known to prefer targets of macromolecular complexes, which are often in the cytoplasm or nucleus, and participate in many cellular processes^35^. Our data demonstrated that the Kac proteins are mainly located in the cytoplasm and nucleus. Histone deacetylase inhibitors, such as MS-27-275, romidepsin and suberoylanilide hydroxamic acid (SAHA) were reported to have anti-tumor activities in cancers, including NB^36^,^37^,^38^,^39^,^40^,^41^. In our protein complex clustering analysis on acetylome data, TNF-α/NF-κB signaling complex 7/8/10, sin3 complex, mSin3A complex, SIN3-HDAC-SAP30-ARID4 complex and BRMS1-SIN3-HDAC complex were enriched in the up-regulated Kac proteins after perifosine treatment. These results were also observed in a lysine acetylome study in NB cells treated with SAHA^42^. Such observations suggested that perifosine and SAHA might share some regulatory mechanisms. Presently, there are very few reports about the acetylation changes induced by perifosine. Future studies will be needed to expose the relationship between perifosine and the acetylation of its target proteins, and whether and how the acetylation affects their activities and functions.

In summary, the present study offers a complete mapping of the proteome and acetylome profile after perifosine treatment in neuroblastoma AS cells. The current study expands our understanding on the mechanism of perifosine effects. Thousands of proteins and hundreds of differentially expressed proteins were screened out from the study. In the future, research on the major target and key pathway may be well worth further validating and exploring in the role of perifosine.

## Methods

### Cells and reagents

SK-N-AS (AS) cell line and SK-N-BE2 (BE2) cell line were used in this study. AS and BE2 cells were cultured in RPMI-1640 medium (Pierce, Rockford, IL, USA) containing 10% fetal bovine serum (Gibco, Grand Island, NY), 100 U/ml penicillin, 100 μg/ml streptomycin and 2 mM/L glutamine and sodium pyruvate at 37 °C in 5% CO_2_ incubator. Perifosine (Selleck Chemicals, Houston, TX, USA) was dissolved in ddH_2_O and stored in −20 °C. Other chemicals such as trifluoroacetic acid (TFA), formic acid (FA), iodoacetamide (IAA), dithiothreitol (DTT) were purchased from Sigma (St. Louis, USA). Trypsin and acetonitrile (ACN) were purchased from Promega (Fitchburg, MI, USA) and Fisher (Waltham, MA, USA) respectively.

### Treatment

To study the response to perifosine, 1.5 × 10^4^ cells were seeded per well with 100 μl medium for 24 h and then treated with vary concentrations of perifosine (2.5, 5, 7.5, 10, 15, 20, 30, 40, 50 and 60 μM) for 48 h in 96-well plates. To determine the effect of perifosine on phosphorylated (P)-Akt, total (T)-Akt and pan acetylation protein, AS cells were seeded at a density of 1.5 × 10^6^ cells/plate in five 100 mm plates for 24 h and treated with vary concentrations of perifosine (2.5, 5, 7.5, 10 and 15 μM) for 16 h, and then harvested and washed twice with ice-cold PBS. To validate the the results from the global proteome and acetylome, AS and BE2 cells were seeded at a density of 1.5 × 10^6^ cells/plate in five 100 mm plates for 24 h and treated with10 μM of perifosine for 16 h, and then harvested and washed twice with ice-cold PBS.

### siRNA and transfection

Integrin β5-siRNA1, integrin β5-siRNA2 and siRNA control were purchased from Ruibo (Guangzhou, China). The sequences of siRNAs were used: integrin β5-siRNA1, sense: 5′-GAGAGAAAUUGGCAGAGAA-3′ and antisense: 3′-CUCUCUUUAACCGUCUCUU-5′; integrin β5-siRNA2, sense: 5′-GAGCCAGAGUGUGGAAACA-3′ and antisense: 3′-CUCGGUCUCACACCUUUGU-5′. AS cells were seeded in 6-well plate, 3 × 10^5^ cells per well. After cultured 24 hours, siRNAs were transfected into cells using jetPRIME (Polyplus Transfection, Illkirsch, France) according to manufacturer’s instructions. After incubation for another 24 hours, cells were used for the MTS assay, wound healing assay and western blotting.

### Cell survival analysis

Cell survival was measured using the MTS assay (3-(4,5-dimethylthiazol-2-yl)-5-(3-carboxymethoxyphenyl)-2-(4-sulfophenyl)-2H-tetrazolium, inner salt assay) according to the manufacturer’s specification. Then, MTS was added to AS cells and incubated at 37 °C in 5% CO_2_ incubator for 2 h afterward. The percentage of cell survival (survival rate) was calculated by normalizing the absorbance value of the treated AS cells by the absorbance value of the control AS cells within each group.

### Western blotting

Protein lysate was extracted and the protein concentration was determined by the BCA method (Biyuntian, Nantong, China). Total 30 μg protein in each condition was loaded onto sodium dodecyl sulfate-polyacrylamide gel electrophoresis (SDS-PAGE) gels, transferred to nitrocellulose membranes (Immobilon-P, Millipore, Bedford, MA, USA). Membranes were blocked with 5% non-fat milk in TBST (10 mM Tris, pH 7.4, 150 mM NaCl and 1‰ Tween-20) at room temperature for 1 h and incubated with the anti-P-Akt (Ser473) antibody (1:1000 dilution, Cell Signaling Technology, Beverly, Mass, USA), anti-T-Akt antibody (1:1000 dilution, Cell Signaling Technology, Beverly, Mass, USA), pan anti-acetyllysine antibody (1:1000 dilution, PTM Biolabs, Hangzhou, China), anti-integrin β5 antibody (1:500 dilution, Abcam, MA, USA), anti-acetyl-Histone H2B (Lys12) antibody (1:2000 dilution, PTM Biolabs, Hangzhou, China), and anti-GAPDH antibody (1:5000 dilution, Kangchen bio-tech, Shanghai, China) at 4 °C overnight. After washing with TBST, the membranes were reacted with the peroxidase-conjugated affiniPure goat anti-rabbit or goat anti-mouse secondary antibodies (1:5000 dilution, Zhongshanjinqiao, Beijing, China) for 1 h at room temperature. After extensive washing with TBST, signals were detected using enhanced chemiluminescent reagents (Thermo Scientific, IL, USA).

### Wound healing assay

Cell migration was measured with wound healing assay. AS cells were seeded into 24-well plates after transfected with siRNA control, integrin β5-siRNA1 and integrin β5-siRNA2 in 48 h. The full layer cells was scratched with a 200 μl pipette tip across the well center after 48 h, then washed with PBS, and cultured with normal media. The wound widths were photographed at 10× magnification at 0 h and 48 h. The beginning and the end wound widths were measured by Image-Pro Plus software respectively. The cell migration rate was calculated as (B − E)/B × 100%, in which B indicates the beginning wound width, and E indicates the end wound width. The experiments were repeated three times.

### SILAC labeling

Either “heavy isotopic lysine” (^13^C-Lysine, ^13^C^15^N-Arginine) or “light isotopic lysine” (^12^C-Lysine, ^12^C^14^N-Arginine) labeled AS cells with a SILAC Protein Quantitation Kit (Pierce, Rockford, IL, USA) in the light of manufacturer’s instructions for over six generations, to attain at least 97% labeling efficiency. Later, the cells continuously grew in SILAC media to desired cell populations (~5 × 10^8^) in fifteen 150 cm^2^ flasks. The “light” and the “heavy” labeled cells were then treated with perifosine and the same amount of DMSO respectively for 16 h. Finally, the cells were harvested and washed twice with ice-cold PBS added with 2 μM trichostatin A and 30 mM nicotinamide.

### Protein extraction and in-solution trypsin digestion

For the whole proteome quantification analysis, the harvested cells were lysed in lysis buffer (8 M urea, 5 μM TSA, 50 mM NAM, 1% PR-619, and 1% cocktail III) on ice for 30 min. For acetylation abundance analysis, the cells were sonicated three times on ice in lysis buffer (8 M Urea, 5 mM DTT, 2 mM EDTA, and 1% cocktail III). After centrifugation at 20,000 g for 10 min at 4 °C, the supernatants were reserved and protein concentrations were measured. Equal amounts of crude proteins in supernatant labeled “heavy” or “light” were mixed and then precipitated by 15% TFA. After washing twice with cold acetone, the protein pellets were resuspended in 100 mM NH_4_HCO_3_ (pH 8.0) and added trypsin at an enzyme-to-substrate ratio (1:50) to digest at 37 °C for 16 h. After that, alkylation reaction was conducted and 5 mM DTT was added for 30 min at 50 °C, followed by 15 mM IAA was added in dark for 30 min at room temperature. Subsequently, 30 mM cysteine was used to quench the alkylation reaction for 30 min at room temperature. Trypsin (ratio of enzyme-to-substrate at 1:100) was then added and incubated for 4 h at 37 °C to accomplete the digestion cycle.

### HPLC fractionation

The peptide mixture derived from tryptic hydrolysis was then separated into fractions by high pH reverse-phase HPLC using Agilent 300 Extend C18 column (5 μm particles, 4.6 mm ID, 250 mm length) with a gradient of 2% to 60% ACN in 10 mM ammonium bicarbonate (pH 10) over 80 min into 80 fractions. Afterward, the peptides were combined into 18 fractions and vacuum dried.

### Affinity enrichment

To enrich Kac peptides, the tryptic peptides were suspended in NETN buffer (100 mM NaCl, 1 mM EDTA, 50 mM Tris-HCl, 0.5% NP-40, and pH 8.0) and incubated with pre-washed anti-acetyllysine antibody beads (PTM Biolabs, Hangzhou, China) overnight with gentle shaking at 4 °C. The peptides-enriched beads were washed four times with NETN buffer and twice with ddH_2_O. Next were the bound peptides eluted from the beads with 0.1% TFA, collected, vacuum dried and cleaned with C18 ZipTips (Millipore) in accordance with the manufacturer’s instructions.

### LC-MS/MS analysis

Peptides were dissolved in 0.1% FA and directly loaded onto a reversed-phase pre-column (Acclaim PepMap 100, Thermo Scientific). Peptide fractions were separated using a reversed-phase analytical column (Acclaim PepMap RSLC, Thermo Scientific). The gradient started at an increase from 6% to 22% solvent B (0.1% FA in 98% ACN) for 26 min, 22% to 35% for 8 min, climbed to 80% in 3 min and maintained at 80% for the last 3 min, all at a constant flow rate of 300 nl/min on an EASY-nLC 1000 UPLC system. The subsequent resulting peptides analysis was performed by Q Exactive^TM^ Plus hybrid quadrupole-Orbitrap mass spectrometer (ThermoFisher Scientific).

The peptides were subjected to NSI source, with subsequent tandem mass spectrometry (MS/MS) in Q Exactive^TM^ Plus (Thermo) coupled online to the UPLC. Intact peptides were detected in the Orbitrap at a resolution of 70,000 and selected for MS/MS with the NCE setting as 30. The fragment ions were detected in the Orbitrap at a resolution of 17,500. A data-dependent procedure that alternated between one MS scan followed by 20 MS/MS scans was applied for the top 20 precursor ions above the ion count threshold of 1E4 with 10 s dynamic exclusion. The electrospray voltage applied was 2 kV. Automatic gain control (AGC) was employed to prevent overfilling of the ion trap. For generation of MS/MS spectra, 5E4 ions were accumulated. The m/z scan range was 350 to 1800 for MS scans.

### Database search

The acquired MS/MS data were processed by MaxQuant with integrated Andromeda search engine (v.1.4.1.2). Tandem mass spectra were searched against SwissProt database (20,203 sequences) concatenated with reverse decoy database. Four missing cleavages, 4 modifications per peptide and 5 charges were allowed for specified trypsin/P as cleavage enzyme. Mass errors of precursor ions and fragment ions were separately set to 10 ppm and 0.02 Da. Carbamidomethylation on Cys was accounted as fixed modification, oxidation on Met, acetylation on Lys and acetylation on protein N-terminal were accounted as variable modifications. False discovery rate (FDR) thresholds for protein, peptide and modification site were set at 1%. Minimum peptide length was set at 7. All the other parameters were default values in MaxQuant. The site localization probability was specified as >0.75.

### Bioinformatic analysis

Gene Ontology (GO) annotation was generated using the UniProt-GOA database (http://www.ebi.ac.uk/GOA/), supplement with the InterProScan soft. The updated version of wolfpsort, PSORT/PSORT II, was used to predict subcellular localization. The protein pathways were annotated from the Kyoto Encyclopedia of Genes and Genomes (KEGG) database. Manually curated CORUM protein complex database for human was selected to analyze protein complex. Protein domain annotation was derived from InterPro domain database. The enrichment analysis was determined by a two-tailed Fisher’s exact test to identity the enrichment of the differential expressed proteins against the background of all identified proteins with a corrected *p* value < 0.05. The differentially expressed proteins involved functional categories were clustered by one-way hierarchical clustering (Euclidean distance, average linkage clustering). To visualize cluster membership, a heat map was carried out using the “heatmap.2” function from the “gplots” R-package. The software motif-x was applied to analysis the amino acid sequence model in specific positions of acetyl-21-mers (Kac site ±10 amino acids) of all identified Kac sequences. The search tool for the Retrieval of Interacting Genes/Proteins (STRING) database was researched to analyze protein-protein interactions of all the identified Kac proteins with a confidence score ≥0.7 (high confidence). The interaction network was visualized by Cytoscape software (version 3.0.1). The detailed description of the bioinformatic analysis was shown in the Supplementary Information.

### Statistical analysis

Comparisons of the cell survival rates and migration rates between two groups were performed using the Student’s t-test. The results were shown as means ± SD.

## Additional Information

**How to cite this article:** Gu, X. *et al*. Proteome and Acetylome Analysis Identifies Novel Pathways and Targets Regulated by Perifosine in Neuroblastoma. *Sci. Rep.* **7**, 42062; doi: 10.1038/srep42062 (2017).

**Publisher's note:** Springer Nature remains neutral with regard to jurisdictional claims in published maps and institutional affiliations.

## Supplementary Material

Supplemental Information

## Figures and Tables

**Figure 1:**
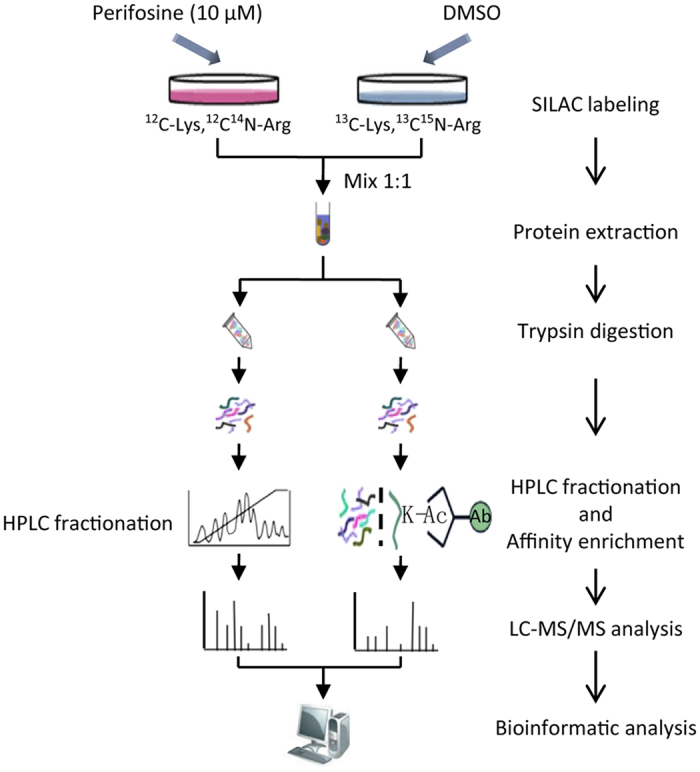
The general technical route for SILAC quantification of the whole proteome and lysine acetylome in perifosine-treated AS cell line.
