# Supplementary material for: Proteome and Acetylome Analysis Identifies Novel Pathways and Targets Regulated by Perifosine in Neuroblastoma
Source: Sci Rep. 2017 Feb 6;7:42062. doi: 10.1038/srep42062 (PMC5292702; doi:10.1038/srep42062)
Supplement: Supplemental Information [file srep42062-s1.pdf]

Supplemental Information

Proteome and Acetylome Analysis Identifies Novel Pathways and Targets Regulated by Perifosine in Neuroblastoma

Xiao Gu[1], Zhongyan Hua[1], Yudi Dong[1], Yue Zhan[1], Xiaowen Zhang[1], Wei Tian[1], Zhihui Liu[2], Carol J Thiele[2], Zhijie Li[1]

[1] Medical Research Center, Shengjing Hospital of China Medical University, Shenyang,
110004, China.

[2] Cellular & Molecular Biology Section, Pediatric Oncology Branch, National Cancer Institute, National Institutes of Health, Bethesda, Maryland, 20892, U.S.A.

Correspondence and requests for materials should be addressed to
Z.J.L. (lizhijie68@hotmail.com, lizj@sj-hospital.org, Ph: +86-18940259465)

Supplemental Methods

Bioinformatic Analysis

KEGG pathway annotation. We used KEGG online service tools KAAS to annotate differentially expressed proteins and then the annotation results were mapped on the KEGG pathway database using KEGG online service tools KEGG mapper.

Motif analysis. All the database protein sequences were used as background parameter, ‘K’ (lysine) was set as modified acid amino “central character”, 21 was set as foreground peptides sequence length “width”, 20 was set as minimal number of peptide occur in one motif “occurrences”, 0.0000001 was set as motif analysis statistics test significance threshold value.

Enrichment-based clustering analysis. After functional enrichment analysis, we collated all the protein groups obtained and their *p* values for further hierarchical clustering based on categories, and then filtered for those categories enriched in at least one of the protein groups with p value < 0.05. This filtered *p* value matrix was transformed by the function $x = -\log10$ (*p* value) and the x values were z-transformed for each functional category. Finally these z scores were clustered by one-way hierarchical clustering (Euclidean distance, average linkage clustering).

Supplemental Figure

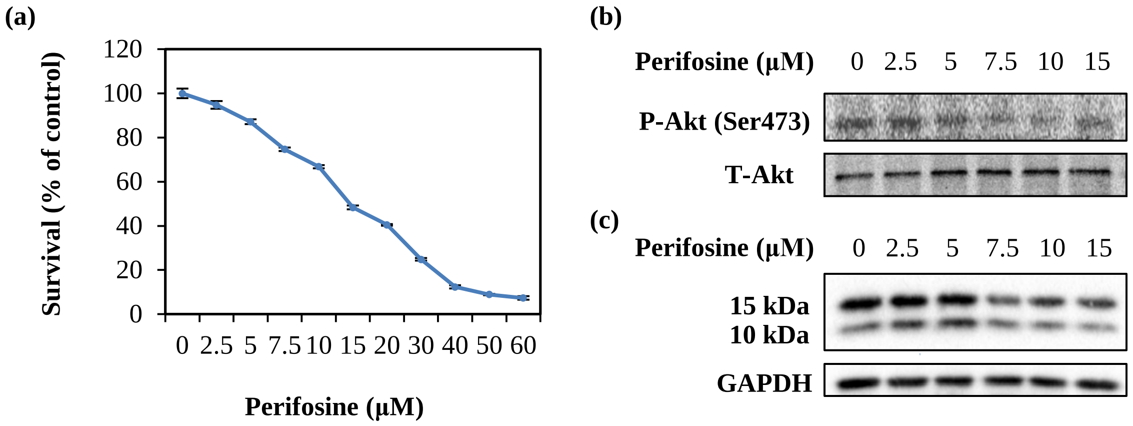

Fig. S1. Effect of perifosine on the survival of AS cells (a), phosphorylated-Akt, total-Akt (b) and pan acetylation protein (c). (**a**) AS cells were treated with different concentrations of perifosine (2.5, 5, 7.5, 10, 15, 20, 30, 40, 50 and 60 µM) for 48 h. MTS assay was used to assess cell survival. Means and standard deviations were shown. (**b**) and (**c**) AS cells were treated with perifosine at different concentrations (2.5, 5, 7.5, 10 and 15 µM) for 16 h. Total proteins were extracted and 30 µg of protein was analyzed for phosphorylated (P)-Akt (Ser473), total (T)-Akt (**b**) and pan acetylation protein (**c**) by western blotting. GAPDH was used as loading control.
